# Region-Specific Biomarkers and Their Mechanisms in the Treatment of Lung Adenocarcinoma: A Study of *Panax quinquefolius* from Wendeng, China

**DOI:** 10.3390/molecules26226829

**Published:** 2021-11-12

**Authors:** Xuanming Zhang, Liwen Han, Peihai Li, Shanshan Zhang, Mengqi Zhang, Xiaobin Li, Jie Chu, Lizhen Wang, Pengfei Tu, Yun Zhang, Kechun Liu

**Affiliations:** 1 Engineering Research Center of Zebrafish Models for Human Diseases and Drug Screening of Shandong Province, Biology Institute, Qilu University of Technology (Shandong Academy of Sciences), Jinan 250103, China; zhangmx@sdas.org (X.Z.); liph@sdas.org (P.L.); zhangss@sdas.org (S.Z.); bjb908@ouc.edu.cn (M.Z.); lixb@sdas.org (X.L.); chuj@sdas.org (J.C.); wanglz@sdas.org (L.W.); 2 Institute of Materia Medica, Shandong First Medical University & Shandong Academy of Medical Sciences, Jinan 256200, China; hanliwen@sdfmu.edu.cn; 3 State Key Laboratory of Natural and Biomimetic Drugs, School of Pharmaceutical Sciences, Peking University, Beijing 100191, China; pengfeitu@bjmu.edu.cn

**Keywords:** *Panax quinquefolius*, biomarkers, network pharmacology, molecular docking, protein phosphorylation

## Abstract

*Panax quinquefolius*, a popular medicinal herb, has been cultivated in China for many years. In this work, the region-specific profiles of metabolites in *P. quinquefolius* from Wendeng was investigated using liquid-chromatography–quadrupole–time-of-flight-(LC–Q–TOF)-based metabolomics analysis. The three most abundant biomarkers, identified as ginsenoside Rb_3_, notoginsenoside R_1_, and ginsenoside Rc, were the representative chemical components employed in the network pharmacology analysis. In addition, molecular docking and western blotting analyses revealed that the three compounds were effective binding ligands with Hsp90α, resulting in the inactivation of SRC and PI3K kinase, which eventually led to the inactivation of the Akt and ERK pathways and lung cancer suppression. The outcomes obtained herein demonstrated the intriguing chemical characteristics and potential functional activities of *P. quinquefolius* from Wendeng.

## 1. Introduction

*Panax quinquefolius* L. (American ginseng) is a well-known traditional herbal medicine with a large demand on the Asian market. Its extracts have also become popular in the US and Europe as dietary health supplements and additives to foods and beverages. The key constituents of the herb include ginsenosides, polysaccharides, peptides, polyacetylenes, and phenols. However, the contents of *P. quinquefolius* are influenced by biological and environmental factors [1,2,3,4,5,6]. Numerous studies have investigated ginsenosides, and the total amount of these natural products (i.e., Rg_1_, Rb_1_, or Re) is used as an index to evaluate the quality of ginseng. Modern pharmacological studies have shown that *P. quinquefolius* plays a role in regulating the immune system and exhibits antitumor, antidiabetic, anti-inflammatory, and antioxidant effects, as well as protective effects on cardiovascular and cerebrovascular systems [7,8,9,10,11,12]. 

*P. quinquefolius* mainly grows in the USA and Canada in the wild and was introduced into China in the last century. In 1964, *P. quinquefolius* was introduced into Wendeng county (122.22° E, 37.25° N), which has become one of the major cultivation areas in China [13]. It is widely recognized that the biological activities of medicinal plants result from the presence of various chemical constituents. Although systematic studies of the components of *P. quinquefolius* have been conducted, and the ages of roots and other parts of the plant have been examined [14,15,16,17,18,19], so far, the medicinal potency of the *P. quinquefolius* cultivated in Wendeng has not been analyzed. Further studies are needed to elucidate the differences in its application and efficacy, despite its overlapping therapeutic indications with the North America species.

Omics-based approaches have been widely employed to study variations in the primary and secondary metabolites in plants. Metabolomics, a relatively new discipline developed after genomics and proteomics, can be used to investigate the changes in the entire metabolic network of an organism and has in recent years attracted considerable attention [20,21,22,23]. Liquid chromatography–mass spectrometry (LC–MS) is a commonly utilized technique in metabolomics-based analysis. Furthermore, network pharmacology is an emerging field, which combines multidimensional information on a “compound–target–disease” and establishes a visualization network to understand the therapeutic effects of a substance [24,25,26]. The above methods can be employed to study the relationships between molecules and diseases. Notably, they can be used simultaneously to investigate active mechanisms based on complex biological systems.

Lung cancer is the most common form of cancer in the world and is the leading cause of cancer-related death. Thus, the development of practical and efficacious therapies for the treatment of the disease is important [27,28]. In this work, metabolomics coupled with network pharmacology was used to evaluate region-specific biomarkers of *P. quinquefolius* from Wendeng. The identified compounds were comprehensively screened and predominantly enriched in cancer-associated pathways, and the mechanisms of the natural products in the treatment of lung adenocarcinoma were then studied. Finally, molecular docking and western blotting analyses were conducted to verify the key targets—i.e., those which exhibited the highest correlation with the disease. 

## 2. Results

### 2.1. Statistical Analysis and Biomarker Discovery

**Figure 1:**
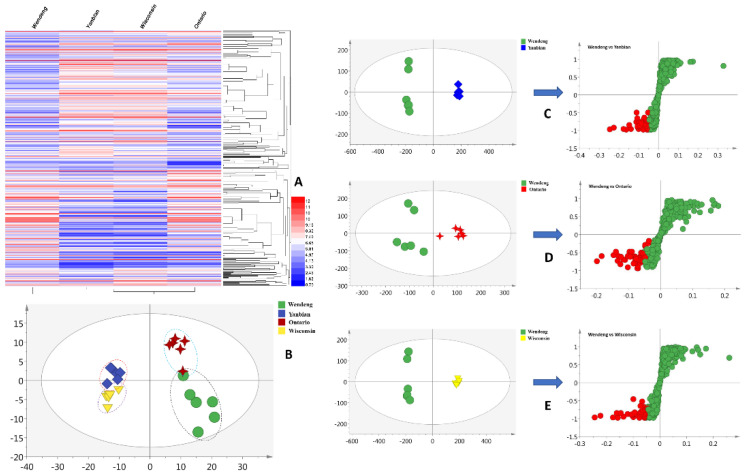
Metabolic profiling of *P. quinquefolius* grown in four different regions. (**A**) Heatmap showing the relative abundance values of all metabolites; (**B**) PCA analysis; (**C**) OPLS-DA plots of the Wendeng and Yanbian species; (**D**) OPLS-DA plots of the Wendeng and Ontario species; (**E**) OPLS-DA plots of the Wendeng and Wisconsin species.

The metabolites of *P. quinquefolius* were investigated using liquid-chromatography–quadrupole–time-of-flight-(LC–Q–TOF)-based methods (Figures S1–S4). A total of 371 primary metabolites were extracted using the R software package in the negative mode. The metabolomic profiles of *P. quinquefolius* grown in Wendeng, Yanbian, Ontario, and Wisconsin are shown as heatmaps in Figure 1A. The obtained heatmaps clearly indicated the relative abundances of the major metabolites in the four groups of samples. To highlight the disparities in the metabolites in the Wendeng species, principal component analysis (PCA) and orthogonal projections to latent structures discriminant analysis (OPLS-DA) were used to classify and discriminate the data. The scores plot yielded a distinct degree of variation between the samples (Figure 1B), suggesting that the metabolic profiles were dissimilar. The values of R^2^ = 0.661 and Q^2^ = 0.517, which represent the quality of fit and predictability, respectively, indicated the reliability of the model [29]. The variables with the VIP values of >1, *p*-values of <0.05, and *p* (corr) of <0.5 were considered to significantly contribute to the separation in the S-plots. Moreover, 42 (Yanbian species), 49 (Ontario species), and 43 (Wisconsin species) metabolites were viewed as potential markers (Figure 1C–E). Furthermore, following omics-based analyses, 30 overlapped metabolites were identified as biomarkers of *P. quinquefolius* cultivated in the Wendeng region (Table S1).

**Figure 2:**
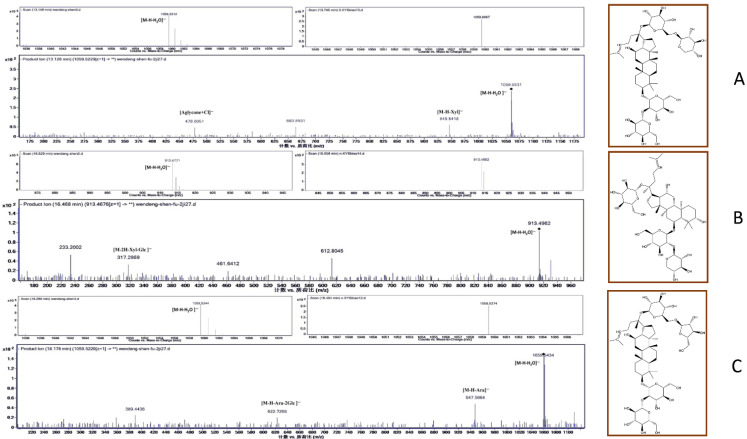
MS and MS/MS spectra of the biomarkers identified in *P. quinquefolius* from Wendeng: (**A**) ginsenoside Rb_3_; (**B**) notoginsenoside R_1_; (**C**) ginsenoside Rc.

The three most abundant biomarkers were identified as ginsenoside Rb_3_ (1, 13.2 min), notoginsenoside R_1_ (2, 16.5 min), and ginsenoside Rc (3, 18.3 min) (Figure 2). In the electrospray ionization–tandem mass spectrometry (ESI–MS/MS) spectra, ginsenoside Rb_3_ exhibited a quasi-molecular ion peak [M − H − H_2_O]^−^ at *m*/*z* 1059.5312 with its daughter ions at *m*/*z* 945 [M − H − Xyl]^−^ and *m*/*z* 478 [Aglycone + Cl]^−^. The spectrum of notoginsenoside R_1_ displayed a quasi-molecular ion peak [M − H − H_2_O]^−^ at *m*/*z* 913.4771 and the corresponding double-charged fragment ion at *m*/*z* 317 [M − 2H − Xyl − Glc]^−^. Lastly, the spectrum of ginsenoside Rc showed a signal corresponding to the [M − H − H_2_O]^−^ ion at *m*/*z* 1059.5344 as the base peak. Additionally, ginsenoside Rc’s fragment ions were detected at *m*/*z* 947.5664 [M − H − Ara]^−^ and *m*/*z* 622.7268 [M − H − Ara − 2Glc]^−^. All peaks were identified through a comparison with the literature data and were further confirmed by the LC–MS analysis of the reference compounds (Figure S5). 

### 2.2. Construction and Analysis of Interaction Networks

**Figure 3:**
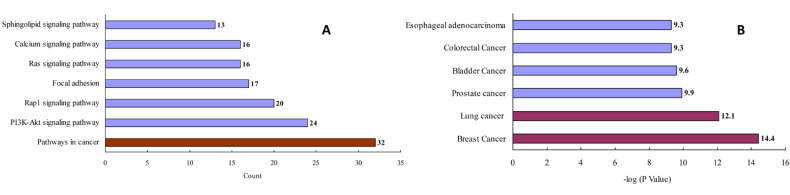
KEGG pathway (**A**) and GAD disease (**B**) enrichment analysis for the analyzed natural products.

After removing redundant entries, 157 protein targets of the three natural products (i.e., ginsenoside Rb_3_, notoginsenoside R_1_, and ginsenoside Rc) were identified and transformed into gene IDs using the Uniprot database (http://www.uniprot.org/, accessed on 2 July 2020). Based on the “count” values, a KEGG enrichment analysis indicated that the top pathway was related to cancers (Figure 3A and Table S2). A GAD disease enrichment analysis revealed that these targets were significantly associated with breast, lung, prostate, bladder, and colorectal cancers, as well as with esophageal adenocarcinoma (Figure 3B).

A total of 274 lung-adenocarcinoma-related genes were obtained using GeneCards to construct a disease-target database. After merging the active compound and disease targets, a PPI network was established by linking the compounds and proteins if they shared one or more target proteins. The visualized network system is illustrated in Figure S6, which suggested the presence of interactions between the molecules and proteins. The degree of each node was defined as the number of edges connected to it and indicated the importance of the node in the network. A topological analysis revealed that HSP90AA1 was an important binding target of the examined compounds. It was determined that HSP90AA1 hit downstream genes SRC, PIK3CA, and PIK3R1 to modulate lung cancer progression (Table S3).

### 2.3. Docking Results Analysis

**Figure 4:**
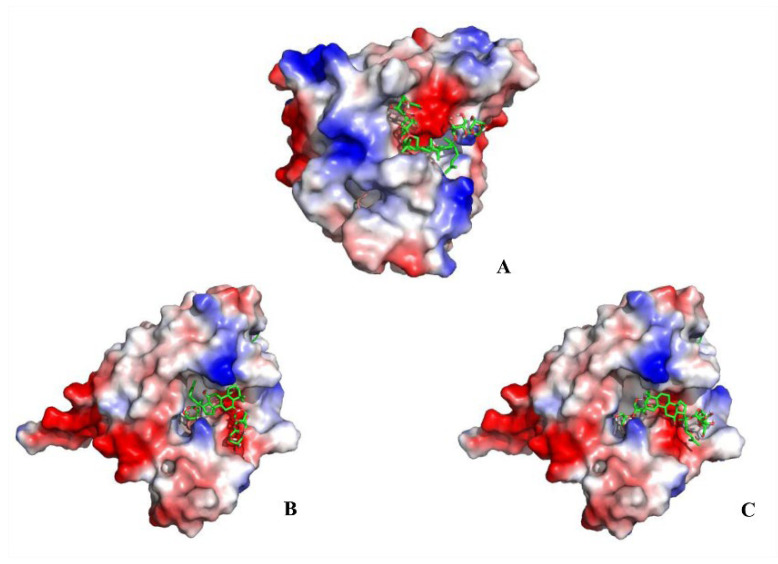
Molecular docking results showing the ligand–receptor interactions between Hsp90α and (**A**) ginsenoside Rb_3_, (**B**) notoginsenoside R_1_, and (**C**) ginsenoside Rc.

Heat shock protein 90α (Hsp90α; HSP90AA1) is a molecular chaperone, which facilitates the correct folding and functionality of its client proteins, predominantly kinases and the nuclear receptors involved in cellular signaling, proliferation, and cell survival. The docking results obtained herein provide a detailed overview of the ligand-receptor interactions. Specifically, ginsenoside Rb_3_, notoginsenoside R_1_, and ginsenoside Rc were confirmed to be effective binding ligands with Hsp90α (Figure 4). Among the three compounds, notoginsenoside R_1_ exhibited the lowest binding free energy (ΔG_b_—3.09 kcal/mol) and formed five hydrogen bonds with the Gly-132 (1.8 Å), Gly-135 (2.8 and 2.4Å), Asn-106 (2.2 Å), and Asp-54 (2.6 Å) residues of the protein. Thus notoginsenoside R_1_ displayed the highest potential affinity with the binding site. The binding energy calculation results for the investigated compounds are summarized in Table S4. Based on the analysis of the molecular interactions, the binding activity of ginsenoside Rb_3_, notoginsenoside R_1_, and ginsenoside Rc with respect to Hsp90α could lead to anticancer activity.

### 2.4. Phosphorylation of SRC, PI3Kα, Akt, and ERK

The steroid receptor coactivator (SRC) is overexpressed in many cancer types. The PIK3CA and PIK3R1 genes encode for phosphoinositide 3-kinase (PI3K) p110α and p85α subunits, which are regulators critical to the cell’s survival. In this study, ginsenoside Rc, ginsenoside Rb_3_, and notoginsenoside R_1_ clearly inhibited the proliferation of the A549 cells, with >50% inhibition rates at 2.5, 2, and 1 mM, respectively (Table 1). To validate our bioinformatics analysis, western blotting was performed to determine the relationships between the compounds and the modified expressions of different proteins in the A549 cells, which led to growth inhibition. 

**Figure 5:**
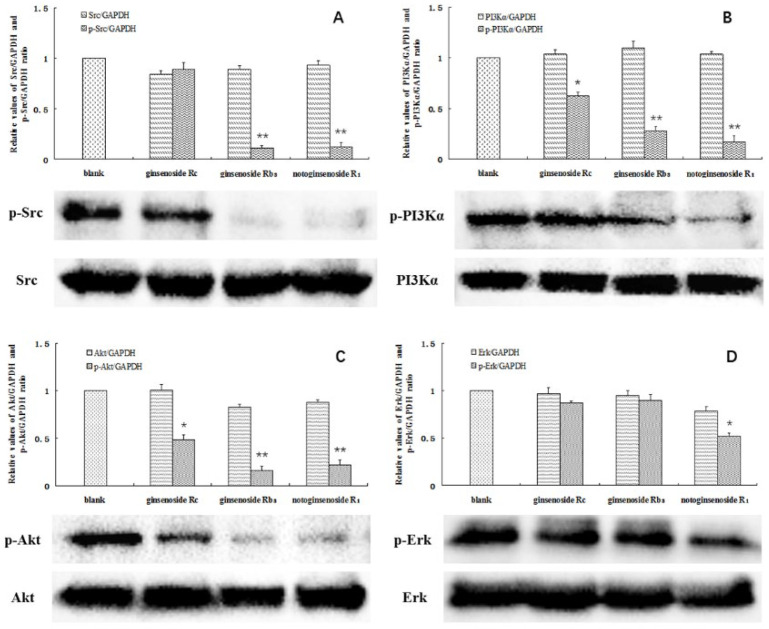
Total and phosphorylated SRC (**A**), PI3Kα (**B**), Akt (**C**), and ERK (**D**) in the A549 cells. Cells without a drug treatment were used as the blank group (relative ratio values were set to 1). * indicates *p* < 0.05, ** indicates *p* < 0.01.

A significant decrease in the protein levels was observed at the above concentrations. The phosphorylation of SRC was almost completely blocked by ginsenoside Rb_3_ and notoginsenoside R_1_. In addition, the phosphorylation of PI3K was significantly inhibited by all three compounds, particularly by ginsenoside Rb_3_ and notoginsenoside R_1_. In contrast, the levels of total SRC and PI3K were not affected (Figure 5A,B). Akt and ERK are the key downstream effectors of SRC and PI3K, and have been considered important factors contributing to the survival of tumor cells. Notably, decreased levels of phosphorylated Akt were found in the A549 cells following treatment with the investigated compounds. Moreover, the ERK phosphorylation was also significantly inhibited by notoginsenoside R_1_ (Figure 5C,D). The acquired data confirms that ginsenoside Rc, ginsenoside Rb_3_, and notoginsenoside R_1_ affected the levels of SRC and PI3K in the cells. Inactivation of SRC and PI3K by the studied compounds affected the downstream Akt and ERK signaling pathways, leading to A549 cell death. 

## 3. Discussion

The present study involved the development of an integrated strategy consisting of metabolomics and network pharmacology to explore region-specific biomarkers and their mechanisms in *P. quinquefolius* cultivated in the Wendeng region. Heatmaps were produced, and PCA and OPLS-DA were conducted to highlight the metabolite disparities between the *P. quinquefolius* originating from four production areas. Ginsenosides were previously recognized as the primary bioactive components in the root of *P. quinquefolius* [30,31]. According to the LC–MS/MS analysis, ginsenoside Rb_3_, notoginsenoside R_1_, and ginsenoside Rc were the most abundant biomarkers in the Wendeng species. Moreover, the KEGG pathway and GAD disease enrichment analyses indicated that the compounds were predominantly enriched in lung-cancer-associated pathways. Potential associations between HSP90AA1 (the drug target) and SRC, PIK3CA, and PIK3R1 (the disease targets) were discovered through integrated network pharmacology. 

Hsp90 inhibitors exert their inhibitory effects by competitively binding at the ATP binding site of the Hsp90 dimer. They interfere with diverse signaling pathways by destabilizing and attenuating the activity of proteins and are selectively toxic to tumor cells [32,33]. Aberrant activation of the Akt pathway is observed in various human cancers. This activation plays a prominent role in various events associated with tumor progression, such as cell proliferation, invasion, migration, and adhesion [34,35,36]. Moreover, the Akt pathway is found to cross talk with the MAPK pathway. Previous studies demonstrated that activated SRC recruits the p85 subunit of PI3K, which results in the activation of PI3K and eventually the activation of Akt and extracellular-signal-regulated kinase (ERK) [37]. Thus, it is speculated that phytochemicals inhibiting the phosphorylation of SRC and PI3K could be promising therapeutics for the treatment of lung cancer. Molecular docking and western blotting results reveal that ginsenoside Rb_3_, notoginsenoside R_1_, and ginsenoside Rc were effective binding ligands with Hsp90α and inactivated SRC and PI3K kinase. The interactions between the compounds and the proteins led to the inhibition of the Akt and ERK signaling pathways, which resulted in tumor cell apoptosis. 

China has a long history of botanical drug use. According to prior reports, *P. quinquefolius* from Wisconsin (USA) and Ontario (Canada) has been recorded as a “genuine regional drug”. Yanbian is the traditional planting region in China. In recent years, Wendeng has become the major *P. quinquefolius* cultivation area in China. The quality requirements of *P. quinquefolius* have been identified in the Chinese Pharmacopoeia. Medicinal herbs that meet the required standards can be used for clinical treatment. However, by comparing the medicinal materials from four different regions, the present study demonstrated the potential of region-specific *P. quinquefolium* components from Wendeng for the treatment of lung adenocarcinoma, which will be helpful for their future use. 

## 4. Materials and Methods

### 4.1. Samples, Chemicals, and Reagents

Four samples of *P. quinquefolius* were obtained from Wendeng (Weihai, China), Yanbian (China), Simcoe, ON, (Canada), and Dane County, WI (USA). The botanical origin of the materials was identified by Professor Kechun Liu, Biology Institute of Shandong Academy of Sciences (Jinan, China). Water (Watsons Ltd., Hong Kong, China) and acetonitrile (Tedia Company Inc., Fairfield, OH, USA) were of LC–MS grade. All other chemicals were of analytical grade. Ginsenoside Rb_3_, notoginsenoside R_1_, and ginsenoside Rc were purchased from Shanghai Yuanye Biotechnology Co., Ltd (Shanghai, China). The A549 cells were acquired from the American Type Culture Collection (ATCC) (Rockville, MD, USA).

### 4.2. LC–MS Assay and Metabolomic Analyses

An amount of 0.5 g of each of the four *P. quinquefolius* samples was accurately weighed and extracted with 5 mL of methanol for 50 min using ultrasound at 50 °C. The solutions were filtered using 0.45 μm millipore filters for subsequent LC–MS analysis. The gradient conditions for LC–MS involved solvents A (water) and B (acetonitrile) in the following ratios: 5–30% B (0–10 min), 30–70% B (10–30 min), 70–100% B (30–40 min), and 100% B (40–45 min). The flow rate was set at 1 mL/min and the injection volume was 20 μL. Sample ionization was performed by electrospray in the negative mode. The following MS conditions were employed: split ratio of 2:1, drying temperature of 350 °C, drying flow of 12 L/min, nebulizer pressure of 40 psi, capillary voltage of 3500 V, and scan range between 200 and 2000 *m*/*z*. The clustering heatmaps were constructed based on the differential metabolites taken from the normalized LC–MS data. A metabolomics method was subsequently used to further investigate the content variation among different groups [38,39]. Variable influence on projection (VIP), p, and p(corr) were applied to identify multiple components with the highest potential as the biomarkers in the OPLS-DA model. Qualitative identification was performed based on high-resolution tandem mass spectrometry (HR-MS/MS) analysis and confirmed by the analysis of standard reference compounds.

### 4.3. Target Database Construction and Bioinformatics Analysis

Ginsenoside Rb_3_, notoginsenoside R_1_, and ginsenoside Rc were used as the representative chemical components of *P. quinquefolius* from Wendeng for network pharmacology analysis. The chemical structures were downloaded from the PubChem database (http://pubchem.ncbi.nlm.nih.gov/, accessed on 30 June 2020). The targets related to the active compounds were screened using Swiss Target Prediction (http://www.swisstargetprediction.ch/, accessed on 30 June 2020). DAVID 6.8 (http://david.abcc.ncifcrf.gov/, accessed on 2 July 2020) was employed for the KEGG pathway and GAD disease enrichment analyses of the targets [40,41]. According to the enrichment scores obtained from the bioinformatics analysis, the target genes associated with the “lung adenocarcinoma” disease name were collected using GeneCards (https://www.genecards.org/, accessed on 2 July 2020). The candidate targets were inputted to String 11.0 (https://string-db.org/, accessed on 2 July 2020) to obtain the relevant information on protein interactions. The active compounds and their potential target proteins were integrated to construct a network employing Cytoscape 3.6.1.

### 4.4. Molecular Docking

Based on the compound–target network construction and analysis, molecular docking was used to confirm the interactions between the ligands and obtained targets. The 3D structures of ginsenoside Rb_3_, notoginsenoside R_1_, and ginsenoside Rc were constructed and stored as PDB files using the PubChem database. The crystallographic structure of the human Hsp90α protein (1.7 Å, HSP90AA1) was downloaded from the Protein Data Bank (PDB) (https://www.rcsb.org/, accessed on 15 July 2020). Each compound was docked into the candidate target using the AutoDock software relying on the genetic algorithm with default parameters [42,43]. Following docking, the affinity scores for the protein–ligand complexes were analyzed, and the results were visualized using the PyMol software [44].

### 4.5. Western Blot Analysis

Apoptosis in A549 cells was evaluated using a 3-(4,5-dimethylthiazol-2-yl)-2,5-diphenyltetrazolium bromide (MTT) assay. The cells were treated with ginsenoside Rb_3_ (2 mM), notoginsenoside R_1_ (1 mM), and ginsenoside Rc (2.5 mM) for 4 h. The cells were then lysed in the RIPA buffer (cat no. P0013B, Beyotime, Shanghai, China), and the expressions of apoptosis-related proteins were evaluated [45,46]. Briefly, the proteins were resolved via 12% sodium dodecyl sulfate-polyacrylamide gel electrophoresis (SDS-PAGE), transferred to polyvinylidene fluoride (PVDF) membranes, and probed with primary antibodies and horseradish peroxidase-conjugated secondary antibodies. The blots were developed using an enhanced chemiluminescence (ECL) kit (cat no. K-12043-D10, Servicebio, Wuhan, China), and the target protein expressions were determined relative to the expression of glyceraldehyde 3-phosphate dehydrogenase (GAPDH).

The following antibodies were used: anti-SRC (1:1000, cat no. #2109, CST, Danvers, MA, USA), anti-p-SRC (1:1000, cat no. #6943, CST), anti-PI3Kα (1:1000, cat no. #4249, CST), anti-p-PI3Kα (1:1000, cat no. #4228, CST), anti-Akt (1:1000, cat no. #4691, CST), anti-p-Akt (1:1000, cat no. #4060, CST), anti-ERK (1:1000, cat no. #4695, CST), anti-p-ERK (1:1000, cat no. #9101, CST), anti-GAPDH (1:5000, cat no. ATPA00013Rb, Atagenix, Wuhan, China), HRP-conjugated goat anti-rabbit IgG (1:5000, cat no. SA00001-2, Proteintech, Rosemont, IL, USA), and HRP-conjugated goat anti-mouse IgG (1:5000, cat no. SA00001-1, Proteintech, Rosemont, IL, USA)

## Figures and Tables

**Table 1:** MTT assay in A549 cells.

Concentrations	Cells Viability		
	Ginsenoside Rc	Ginsenoside Rb_3_	Notoginsenoside R_1_
0.1 mM	92.05%	87.24%	86.40%
0.5 mM	91.41%	78.58%	65.91%
1 mM	75.33%	72.22%	42.73%
1.5 mM	72.04%	64.27%	14.24%
2 mM	59.84%	43.01%	4.52%
2.5 mM	49.18%	24.11%	1.50%
3 mM	40.53%	1.66%	0.61%

## Data Availability

Not applicable.

## Supplementary Materials

The following are available online: Figure S1, LC–Q–TOF-MS chromatograms of *P. quinquefolius* from Wendeng (six samples); Figure S2, LC–Q–TOF-MS chromatograms of *P. quinquefolius* from Yanbian (six samples); Figure S3, LC–Q–TOF-MS chromatograms of *P. quinquefolius* from Ontario (six samples); Figure S4, LC–Q–TOF-MS chromatograms of *P. quinquefolius* from Wisconsin (six samples); Figure S5, The retention times for the three biomarkers in Wendeng species (1) Ginsenoside Rb_3_ (2) Notoginsenoside R_1_ (3) Ginsenoside Rc; Figure S6, The compound-target network. The yellow node represents the most important targets. (Potential compounds: blue rectangles; Drug targets: green triangles; Disease targets: brown circles). Table S1, 30 biomarkers of *P. quinquefolius* from the Wendeng region in OPLS-DA analysis; Table S2, KEGG pathway information for the three compounds; Table S3, Topological parameters for the key targets; Table S4, Binding energy calculation results.
