# Supplementary material for: Region-Specific Biomarkers and Their Mechanisms in the Treatment of Lung Adenocarcinoma: A Study of Panax quinquefolius from Wendeng, China
Source: Molecules. 2021 Nov 12;26(22):6829. doi: 10.3390/molecules26226829 (PMC8623508; doi:10.3390/molecules26226829)
Supplement: Supplementary file 1 [file molecules-26-06829-s001.zip › molecules-1392931-supplementary.pdf]

Supporting information

Region-specific Biomarkers and Their Mechanisms in the Treatment of Lung Adenocarcinoma: A Study of *Panax quinquefolius* from Wendeng, China

Xuanming Zhang [a], Liwen Han [b], Peihai Li [a], Shanshan Zhang[a], Mengqi Zhang [a], Xiaobin Li [a], Jie Chu [a], Lizhen Wang [a], Pengfei Tu [c], Yun Zhang [a, *], Kechun Liu [a, *]

[a] *Engineering Research Center of Zebrafish Models for Human Diseases and Drug Screening of Shandong Province, Biology Institute, Qilu University of Technology (Shandong Academy of Sciences), Jinan 250103, China.*

[b] *Institute of Materia Medica, Shandong First Medical University & Shandong Academy of Medical Sciences, Jinan 256200, China*

[c] *State Key Laboratory of Natural and Biomimetic Drugs, School of Pharmaceutical Sciences, Peking University, Beijing 100191, China.*

*Corresponding author: Kechun Liu, hliukch@sdas.org; Yun Zhang, zhangyun@sdas.org

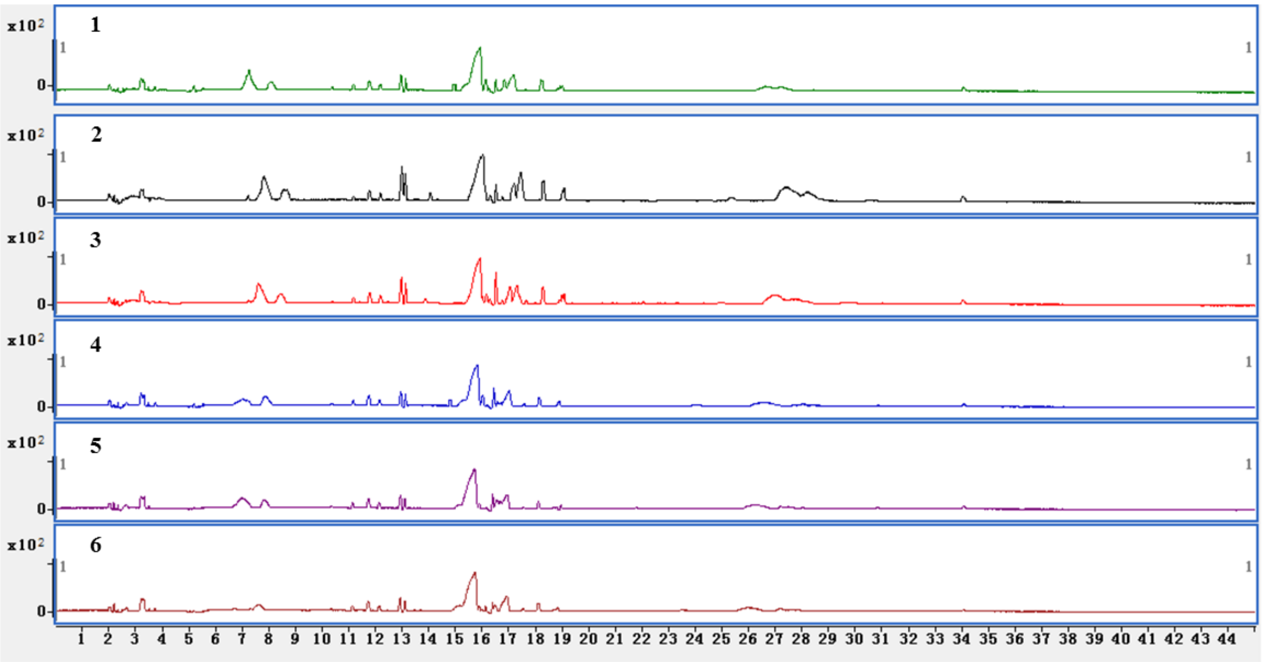

Fig. S1 LC-Q/TOF-MS chromatograms of *P. quinquefolius* from Wendeng (six samples)

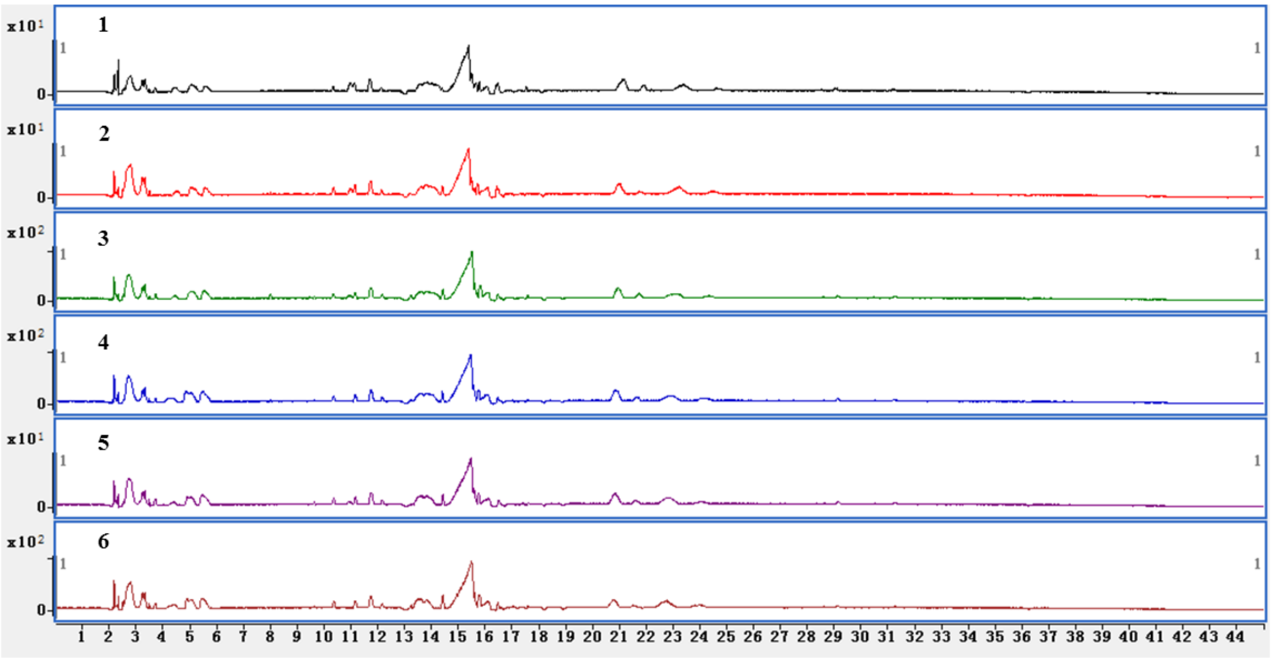

Fig. S2 LC-Q/TOF-MS chromatograms of *P. quinquefolius* from Yanbian (six samples)

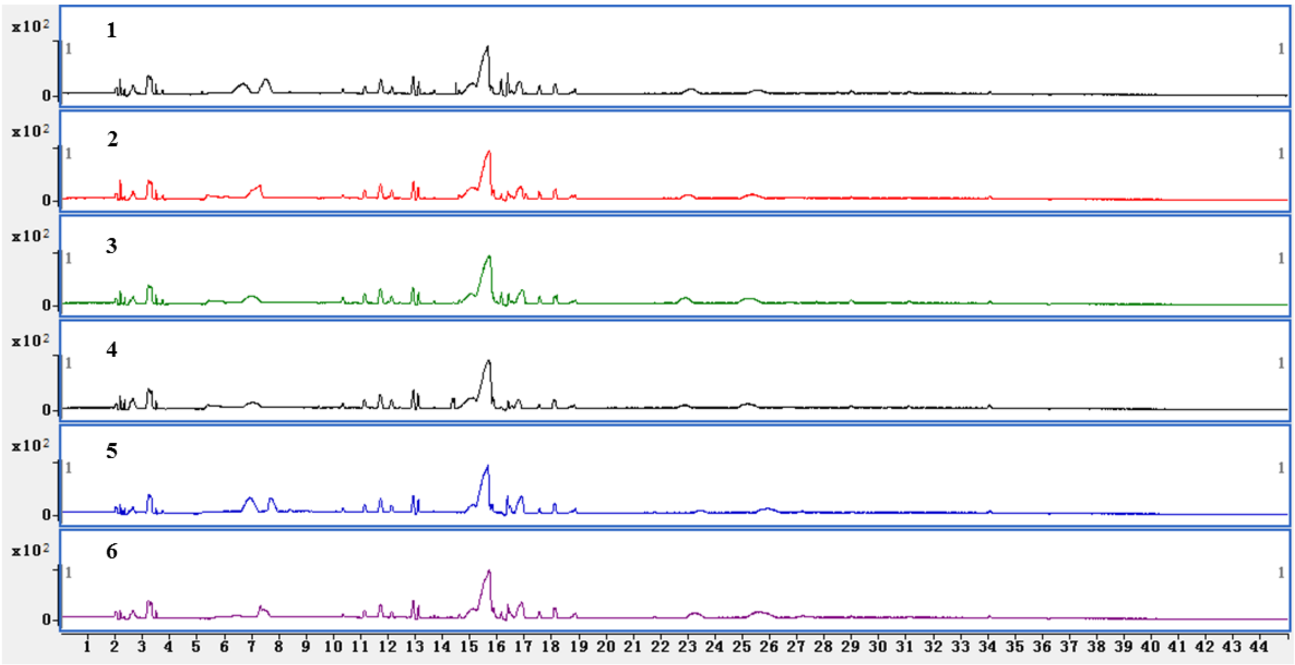

Fig. S3 LC-Q/TOF-MS chromatograms of *P. quinquefolius* from Ontario (six samples)

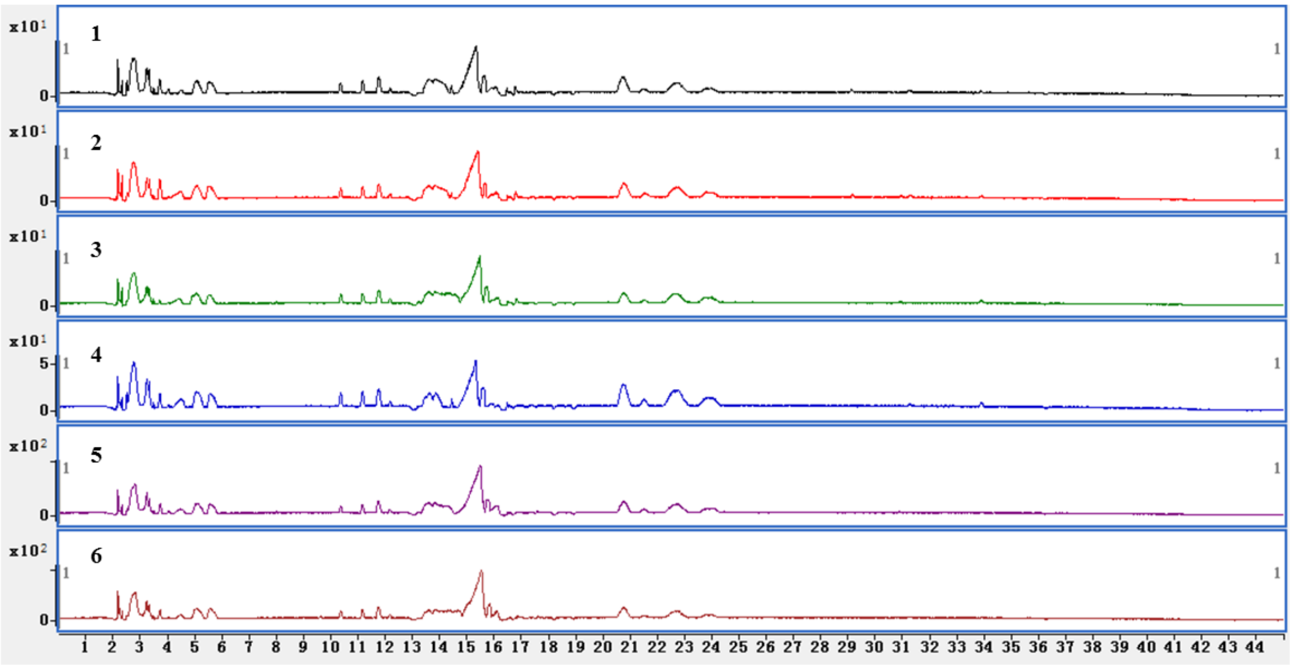

Fig. S4 LC-Q/TOF-MS chromatograms of *P. quinquefolius* from Wisconsin (six samples)

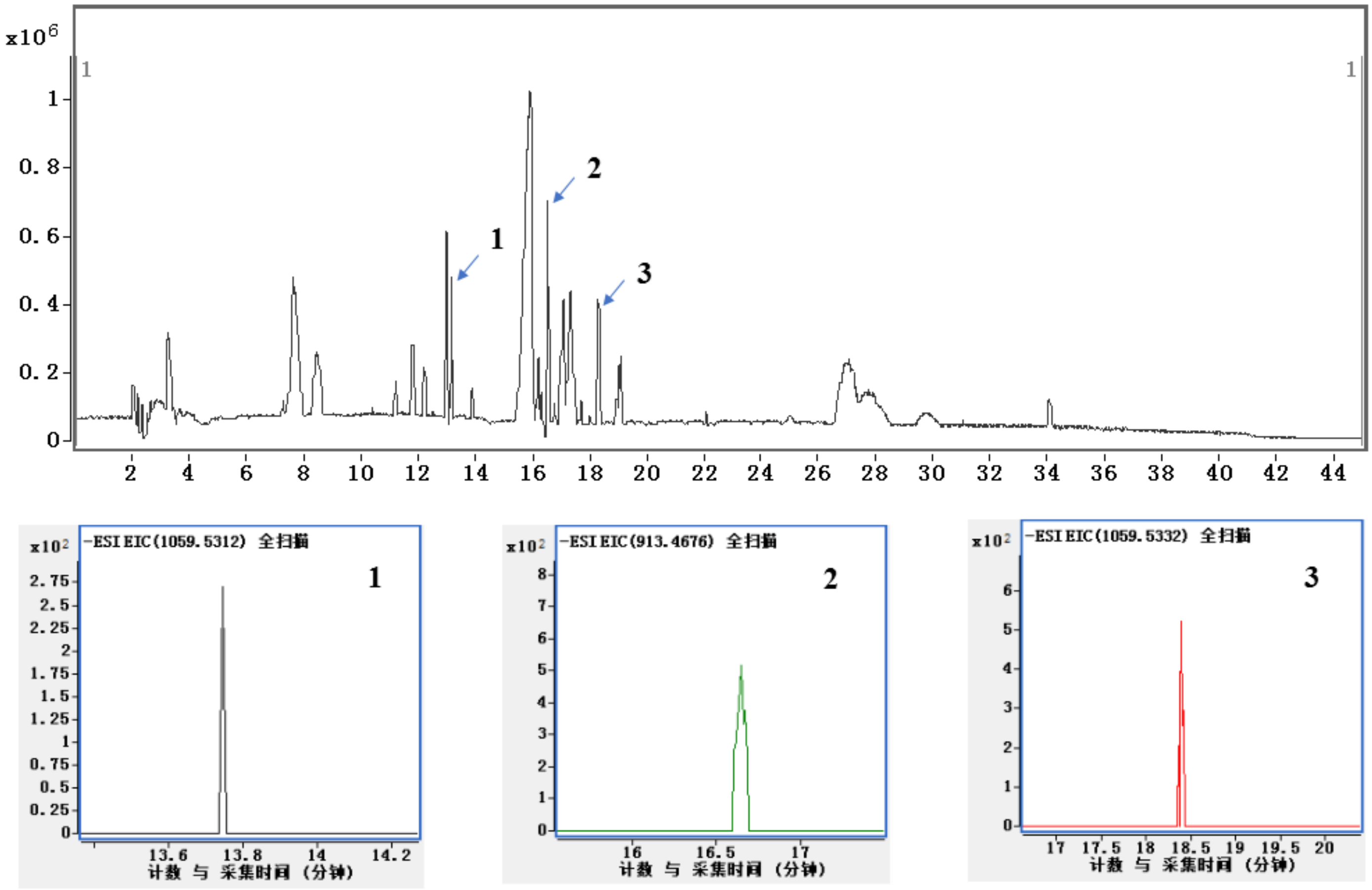

Fig. S5 The retention times for the three biomarkers in Wendeng species. (1) Ginsenoside Rb_3; (2) Notoginsenoside R_1; (3) Ginsenoside Rc.

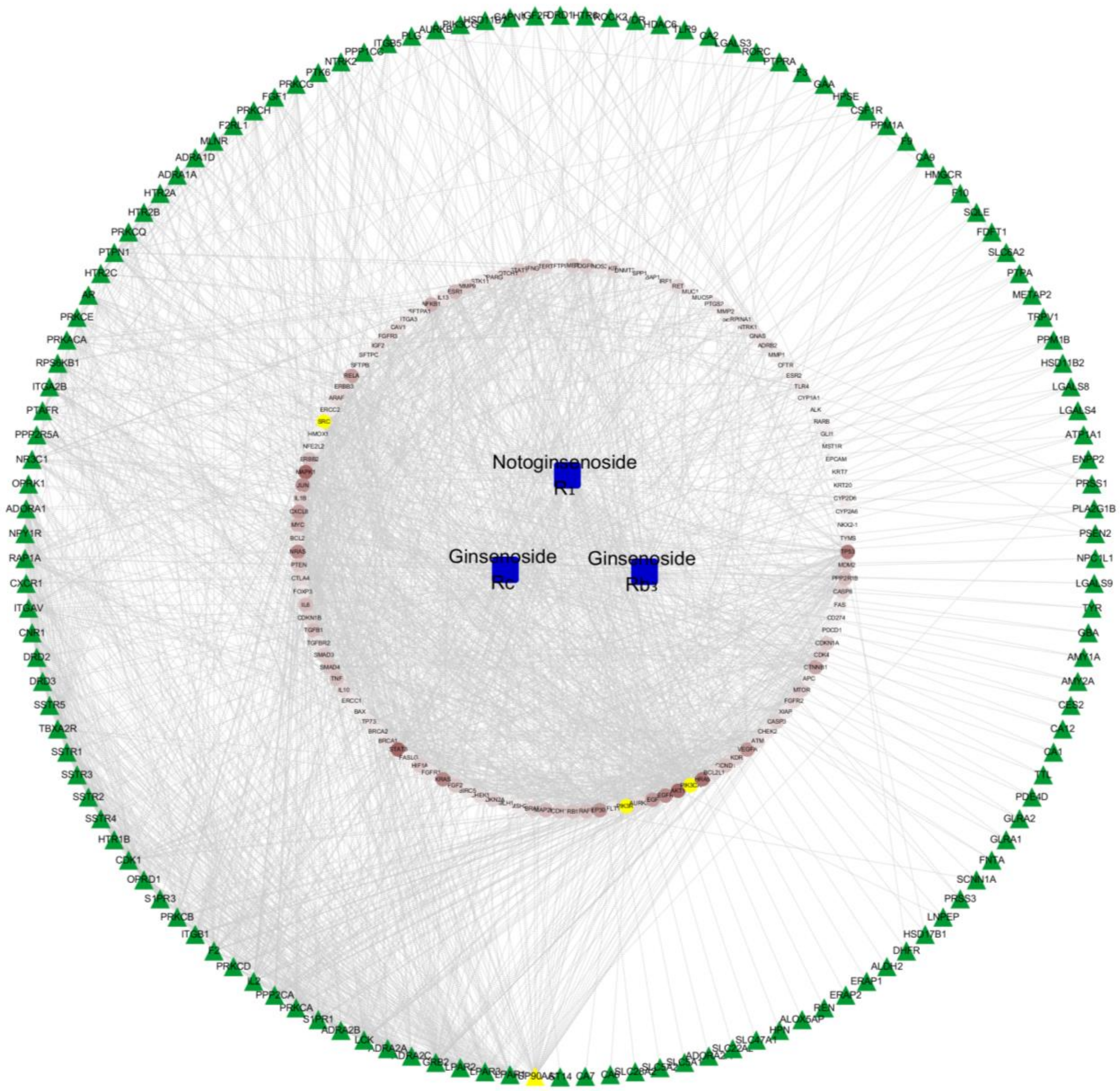

Fig. S6 The compound-target network. The yellow node represents the most important targets. (Potential compounds: blue rectangles; Drug targets: green triangles; Disease targets: brown circles)

Table S1. 30 biomarkers of *P. quinquefolius* from Wendeng region in OPLS-DA analysis

Peak	RT (min)	m/z	VIP	p	p(corr)	Normalized abundances
1	3.25	683.2232	2.22746	-0.11194	-0.7594	2819.36
2	5.51	446.1504	1.15009	-0.06126	-0.6936	362.94
3	7.29	203.0813	1.85684	-0.09925	-0.9353	1247.83
4	11.07	1007.5399	1.02286	-0.05415	-0.9501	219.65
5	11.75	723.4994	1.43273	-0.07598	-0.9722	455.94
6	12.16	836.5832	1.22344	-0.06167	-0.8683	322.95
7	12.16	826.5544	1.10463	-0.05867	-0.9308	860.49
8	13.03	1060.5364	3.36435	-0.17709	-0.9613	2515.41
9	13.03	1009.5385	2.05975	-0.10471	-0.8868	910.89
10	13.03	1061.5384	1.90247	-0.09981	-0.9553	810.91
11	13.04	1059.5332	4.65951	-0.24595	-0.9682	4776.59
12	13.10	1008.5352	2.05542	-0.10612	-0.9136	911.63
13	13.72	845.4869	1.09997	-0.05847	-0.9766	249.44
14	14.65	887.4975	1.0744	-0.05667	-0.9285	253.15
15	16.26	1170.587	1.32096	-0.06055	-0.7350	431.03
16	16.26	1171.5901	1.02424	-0.04592	-0.6967	239.81
17	16.26	1221.5849	1.82219	-0.08709	-0.7931	890.19
18	16.26	1222.5881	1.43611	-0.06915	-0.8036	539.23
19	16.26	1107.5913	1.06147	-0.05358	-0.8469	265.60
20	16.44	914.4782	1.83582	-0.09614	-0.7620	1384.49
21	16.44	863.4803	1.11939	-0.05653	-0.7574	433.87
22	16.45	862.4775	1.55513	-0.07739	-0.71502	985.12
23	16.46	913.4749	2.09314	-0.10758	-0.65398	2982.39
24	16.50	845.4873	1.07698	-0.05265	-0.73603	268.57
25	16.66	1140.5767	1.42603	-0.07039	-0.82814	427.49
26	16.73	1141.5801	1.00509	-0.04547	-0.70246	212.66
27	18.15	1059.5332	4.32288	-0.22588	-0.92351	4017.21
28	18.21	1060.5364	2.94196	-0.1483	-0.84331	1906.08
29	22.01	897.4797	1.44797	-0.07219	-0.63376	579.39
30	34.08	295.2262	3.06384	-0.1637	-0.9768	1954.37

*The biomarkers 11, 23 and 27 with the highest abundances were identified as Ginsenoside Rb_3 (**1**), Notoginsenoside R_1(**2**) and Ginsenoside Rc (**3**), respectively.

Table S2. KEGG Pathway information for the three compounds

KEGG Pathway	Count	Target
Pathways in cancer	32	BCL2L1, GLI1, JUN, MET, ROCK2, AR, CSF1R, FGF1, FGF2, GRB2, HSP90AA1, HIF1A, ITGA2B, ITGAV, ITGB1, LPAR1, LPAR2, LPAR3, MMP9, MTOR, NTRK1, NOS2, PIK3CA, PIK3CG, PTGS2, PRKCA, PRKCB, PRKCG, PRKACA, RET, STAT3, VEGFA
PI3K-Akt signaling pathway	24	BCL2L1, MET, CSF1R, FGF1, FGF2, GRB2, HSP90AA1, ITGA2B, ITGAV, ITGB1, ITGB5, IL2, KDR, LPAR1, LPAR2, LPAR3, MTOR, PIK3CA, PIK3CG, PRKCA, PPP2CA, PPP2R5A, RPS6KB1, VEGFA
Rap1 signaling pathway	20	MET, RAP1A, ADORA2A, CNR1, CSF1R, DRD2, FGF1, FGF2, ITGA2B, ITGB1, KDR, LPAR1, LPAR2, LPAR3, PIK3CA, PIK3CG, PRKCA, PRKCB, PRKCG, VEGFA
Focal adhesion	17	JUN, MET, RAP1A, ROCK2, GRB2, ITGA2B, ITGAV, ITGB1, ITGB5, KDR, PIK3CA, PIK3CG, PRKCA, PRKCB, PRKCG, PPP1CC, VEGFA
Ras signaling pathway	16	BCL2L1, MET, RAP1A, CSF1R, FGF1, FGF2, GRB2, KDR, PIK3CA, PIK3CG, PLA2G1B, PRKCA, PRKCB, PRKCG, PRKACA, VEGFA
Calcium signaling pathway	16	HTR2A, HTR2B, HTR2C, HTR6, ADORA2A, ADRA1A, ADRA1D, ADRB2, DRD1, NOS2, PTAFR, PRKCA, PRKCB, PRKCG, PRKACA, TBXA2R
Sphingolipid signaling pathway	13	ROCK2, ADORA1, OPRD1, PIK3CA, PIK3CG, PRKCA, PRKCB, PRKCE, PRKCG, PPP2CA, PPP2R5A, S1PR1, S1PR3

Table S3. Topological parameters for the key targets

**Description**	**Gene code**	**Degree**	**Betweenness**	**Closeness**
drug-target	HSP90AA1	46	0.029	0.528
disease-target	SRC	61	0.023	0.489
disease-target	PIK3CA	74	0.043	0.534
disease-target	PIK3R1	69	0.025	0.487

Table S4. Binding energy calculation results

Compound	Binding energy ΔG_b (kcal/mol)	Intermolecular energy (kcal/mol)	Electrostatic energy (kcal/mol)	Unbound energy (kcal/mol)
Ginsenoside Rb_3	−1.19	−8.94	−0.39	−9.55
Notoginsenoside R_1	−3.09	−9.36	−0.22	−12.32
Ginsenoside Rc	−0.68	−8.74	−0.48	−11.96
